# Supplementary material for: The precursor of PI(3,4,5)P3 alleviates aging by activating daf-18(Pten) and independent of daf-16
Source: Nat Commun. 2020 Sep 8;11:4496. doi: 10.1038/s41467-020-18280-4 (PMC7479145; doi:10.1038/s41467-020-18280-4)
Supplement: Supplementary file 1 — Supplementary Information [file 41467_2020_18280_MOESM1_ESM.pdf]

Supplementary Information

The precursor of PI(3,4,5)P_3 alleviates aging by activating *daf-18*(*Pten*) and independent of *daf-16*

Shi, Xia, Cui et al.

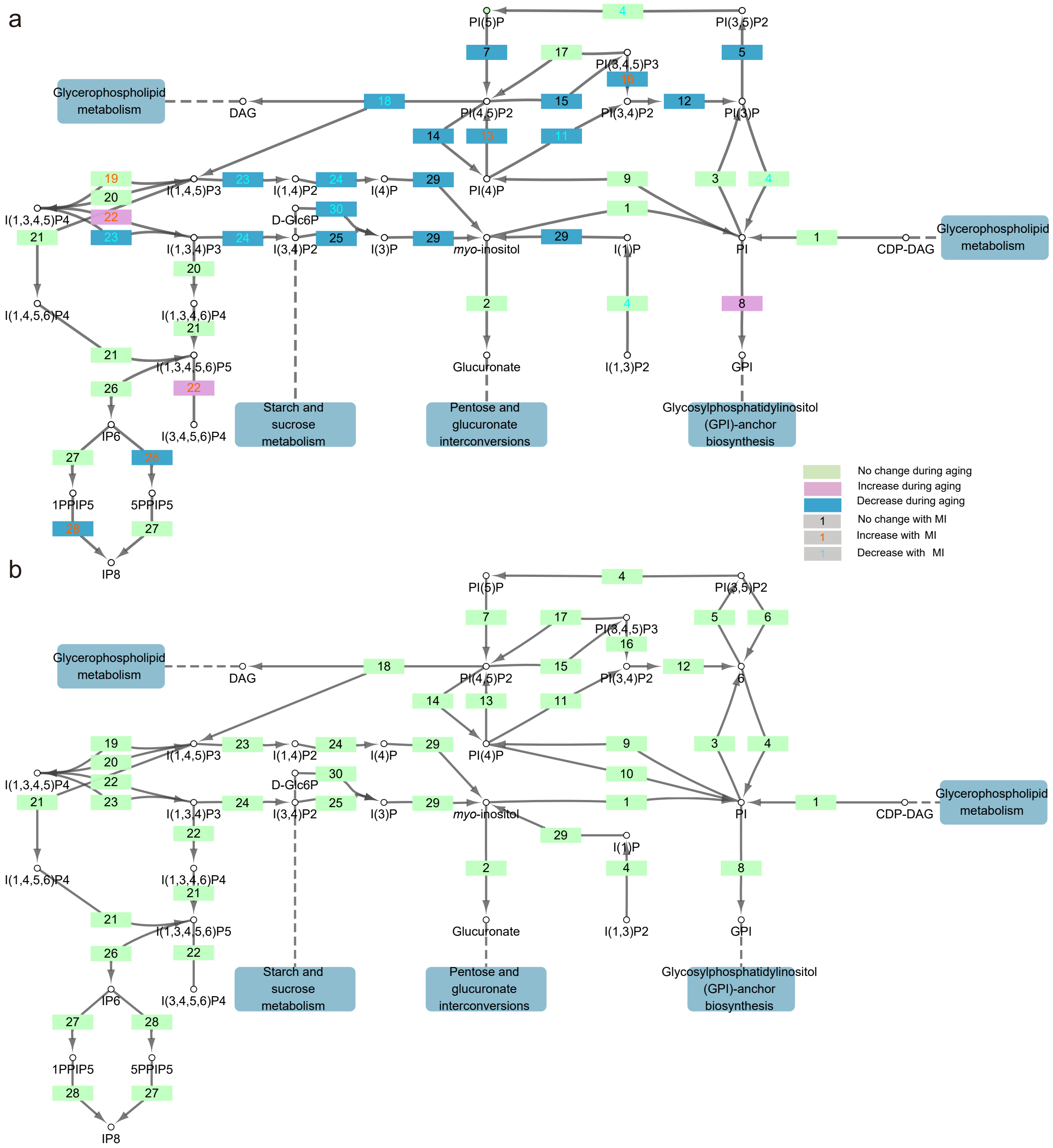

Supplementary Figure 1 | MI metabolic pathway in *Mus musculus* (a) and *Homo sapiens* (b). The arrow head shows the reaction direction. The number labels the reaction step, which can be catalyzed by one or more enzymes. In panel a, the color of enzyme node shows the mRNA increased (plum) or decreased (blue) with age in muscle. The label color of the reaction number indicates MI supplement increased (purple) or decreased (cyan) the enzymes' mRNA levels in muscle. Only results with $\log_2$-fold-change>0.26 are labeled here, N=1.

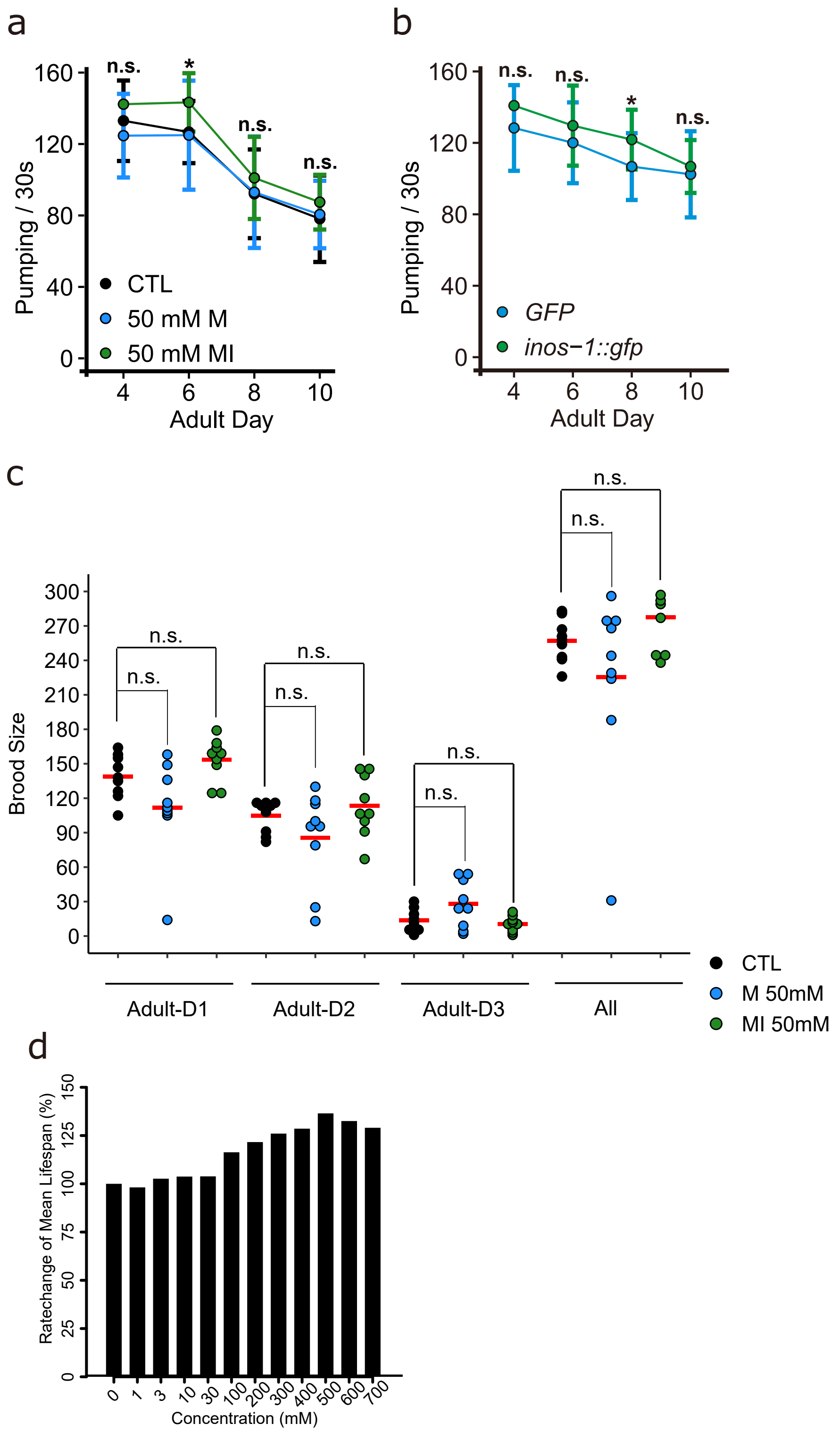

Supplementary Figure 2 | MI alleviates the decline of worm pumping rate during aging. a, 50mM MI alleviated the decrease of worm pumping rate from AD_4 to AD_10 (Data are presented as mean values ± SEM, n=10, two-sided t-test * *p*<0.05 on AD_6), N=2. **b**, *inos-1* OE alleviated the decrease of worm pumping rate from AD_4 to AD_10. (Data are presented as mean values ± SEM, n=10, two-sided t-test * *p*<0.05 on AD_6), N=2. **c**, MI did not influence worm brood size, when treated from L4 stage to AD_3 (n=9, two-sided t-test n.s. not significant), N=2. **d**, Lifespan compared to control (100%) by MI at dosages from 1 mM to 700mM (under 100uM of FUdR), N=2. Source data are provided as a Source Data file.

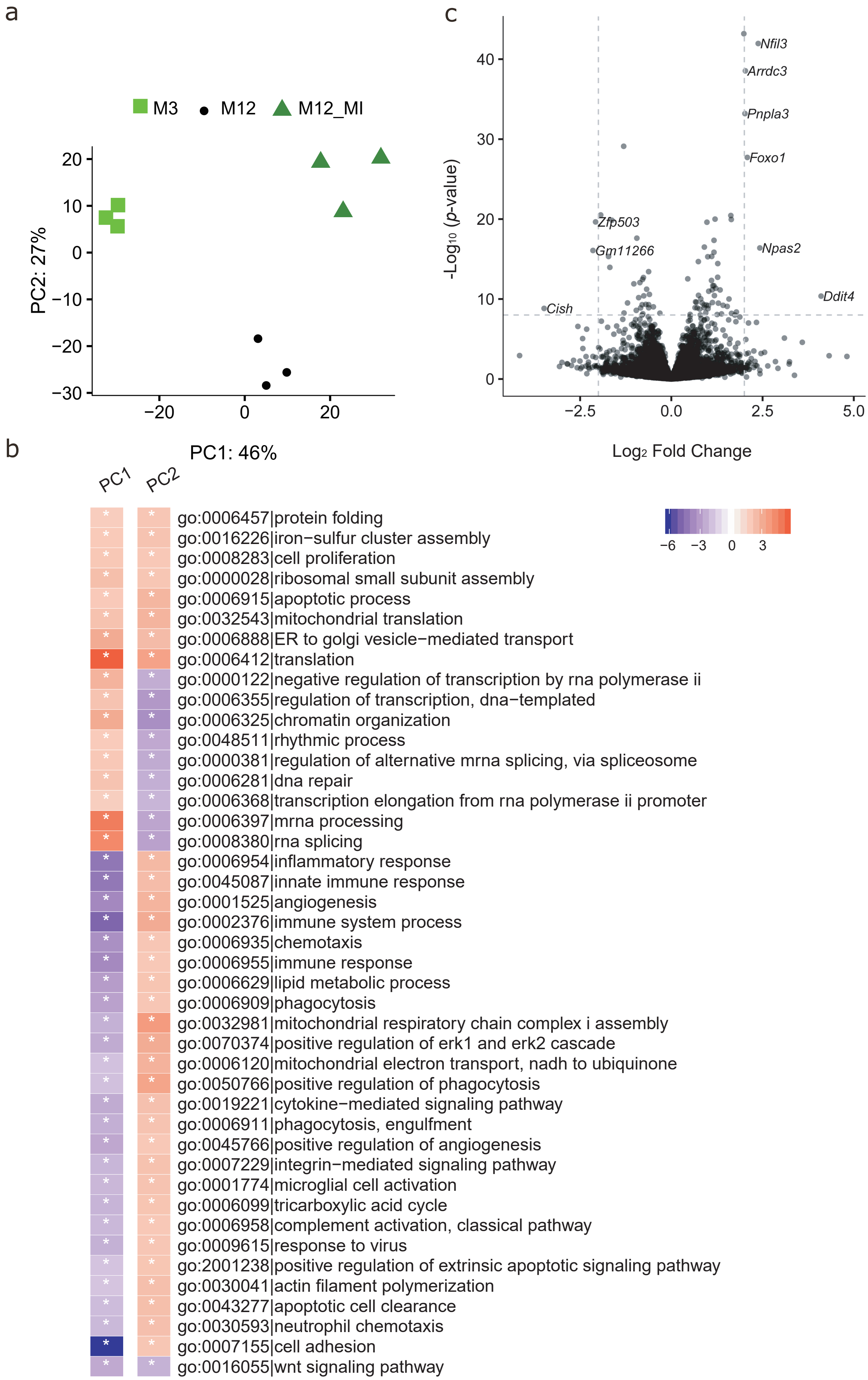

Supplementary Figure 3 | Analysis of mouse transcriptome data. a , PCA analysis of the 9 samples (PC1=46%, PC2=27%) using all expressed genes. **b**, GO analysis of PC1 and PC2 genes. Gene set enrichment analysis (GSEA) was used with, on genes ranked by PCA loadings on PC1 and PC2, respectively. **c**, Volcano plot shows the up-regulated or down-regulated genes after MI treatment, as compared to CTL samples. Source data are provided in Supplementary Dataset 2.

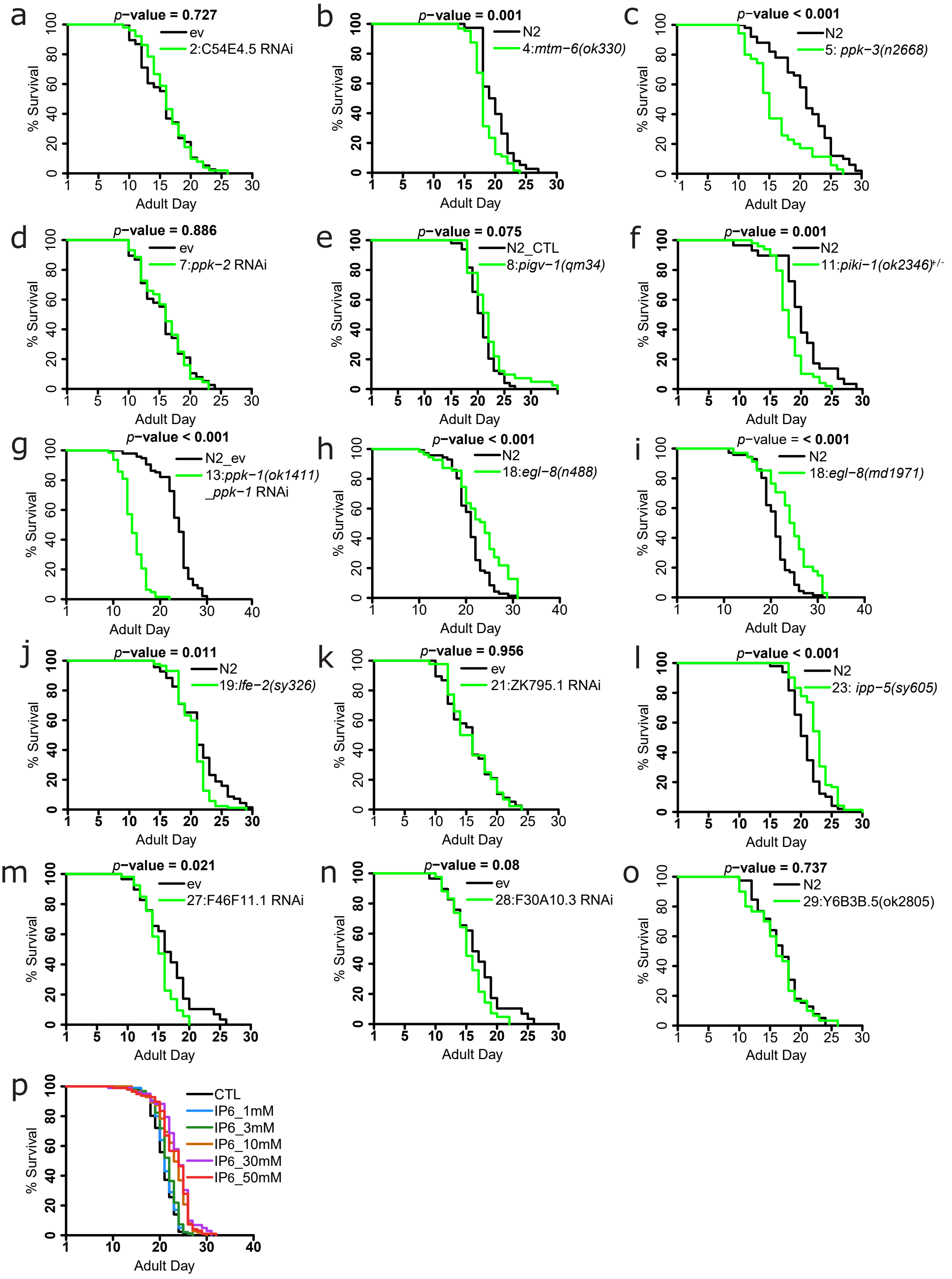

Supplementary Figure 4 | Survival curves of worms with LOF enzymes in MI metabolic pathway. **a - o**, Survival curves of mutant or RNAi worms with LOF or decreased MI metabolic enzymes and their respective controls (see Supplementary Table 3 for detailed data for all survival curves). *p*-value was calculated with log-rank-test. The number indicates the reaction step in Fig 4a (a, N=1; b, N=2; c, N=2; d, N=2; e, N=3; f, N=2; g, N=3; h, N=2; i, N=2; j, N=2; k, N=2; l, N=5; m, N=2; n, N=2; o, N=2). **p**, Survival curves of N2 worms fed with indicated concentrations of IP_6 and control untreated N2 worms, N=2. Source data are provided as a Source Data file.

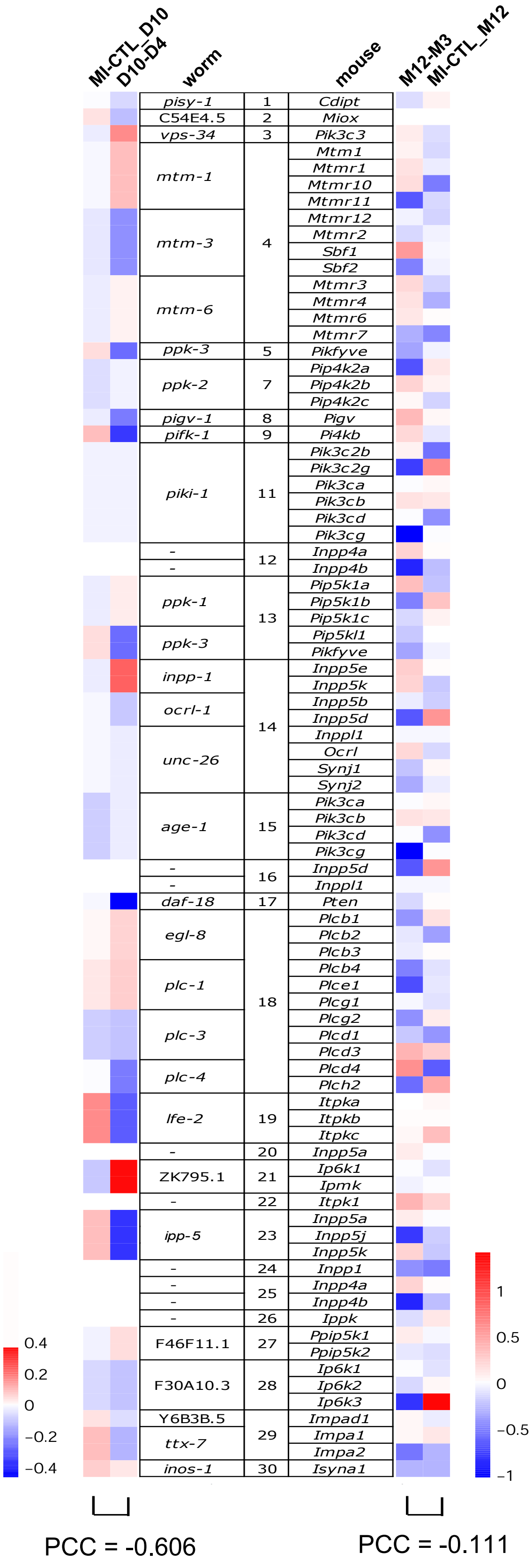

Supplementary Figure 5 | Gene expression changes of MI metabolic enzymes in worm and mouse muscle during aging and upon MI treatment. a, Worm AD_4 and AD_10, or Month-3 and Month-12 of mouse muscle were compared as aging related changes. MI vs. CTL indicates the change between MI treatment and CTL. PCC between D10-D4 and MI-CTL_D10 is -0.606, PCC between M12-M3 and MI-CTL_M12 is -0.111.

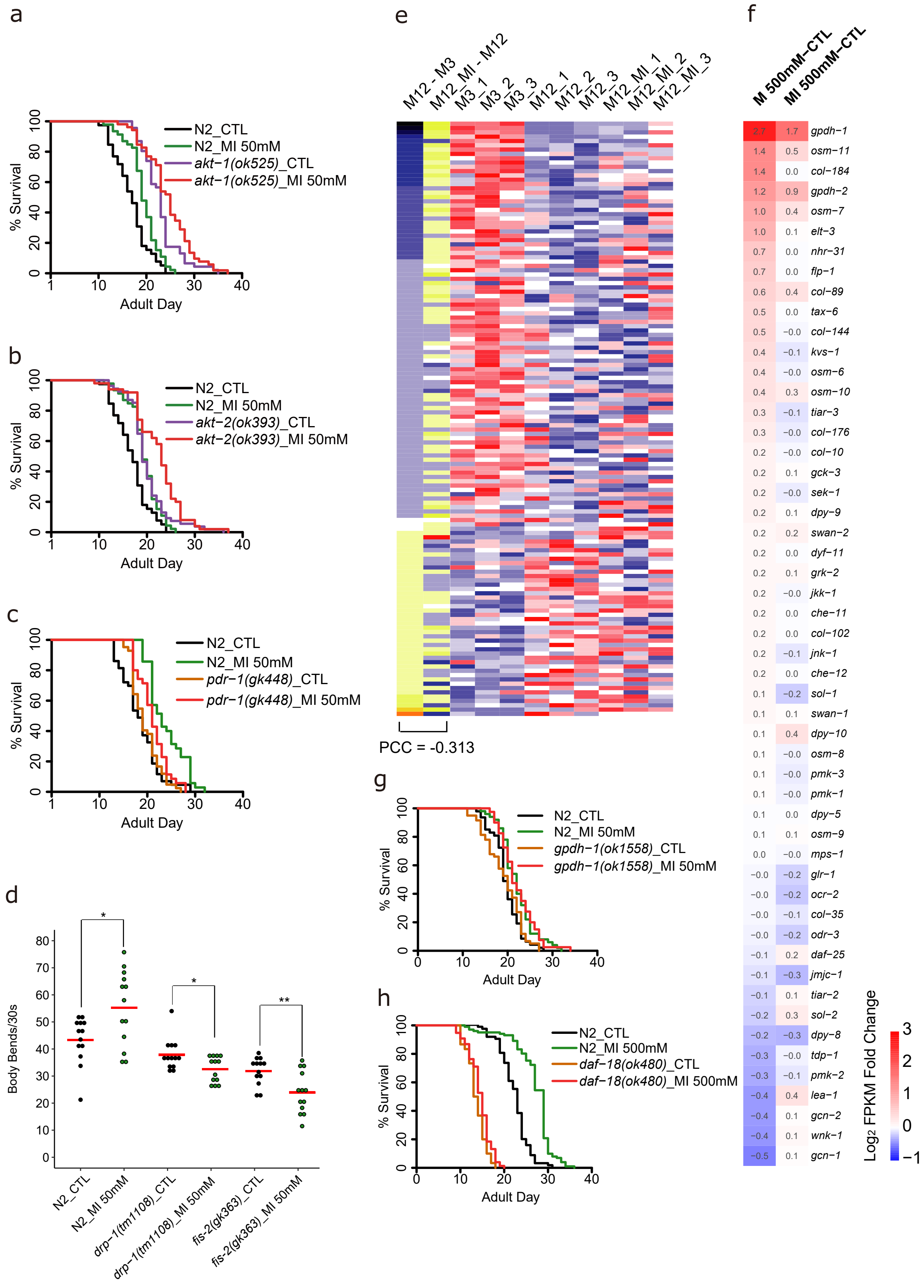

Supplementary Figure 6 | Additional mechanistic analyses of the MI effect. a, **b**, Survival curves of N2 or *akt-1*-LOF, *akt-2*-LOF worms with or without MI treatment, N=2. **c**, Survival curves of N2 or *pdr-1*-LOF worms with or without MI treatment, N=2. **d**, *drp-1* and *fis-2* mutants could block the mobility promoting effect of MI seen in control N2 worms (n=13 in each group, Data are presented as mean values, two-sided t-test * *p*<0.05, ** *p*<0.01), N=2. **e**, Clustering of mitochondria-related genes (PCC= -0.313 between M12-M3 and M12_MI-M12). **f**, Heatmap shows the change of osmotic related genes in mRNA level as determined by RNA-seq log_2-fold-change in FPKM, upon 500mM M or MI treatment for 10 days from AD_1 compared to control on AD_10. **g**, Survival curves of N2 or *gpdh-1*-NL worms with or without 50 mM MI, N=2. **h**, Survival curves of N2 or *daf-18*-NL worms with or without 500 mM MI, N=7. Source data are provided as a Source Data file.`

Supplementary Table 1

Worm strains	From
AC257(*ppk-3(n2668)*)	CGC
AM141(*rmIs133 [unc-54p::Q40::yfp]*)	Prof. Martin S. Denzel
CF1903(*glp-1(e2141)*)	CGC
DA2123（*adIs2122 [lgg-1p::gfp::lgg-1 + rol-6(su1006)*])	CGC
DCL4(*rsks-1(OK1255)*)	Prof. Di Chen Lab
EG3027(*unc-26(s1710)*)	CGC
GR1307(*daf-16(mgDf50)*)	CGC
HZ1770(*bpIs287[Pmyo-3::sqst-1::gfp]*)	Prof. Hong Zhang Lab
IK575((*ttx-7(nj40))*	CGC
IK589(*ttx-7(nj50))*	CGC
IK591(*ttx-7(nj51))*	CGC
IK777(*egl-8(nj77)*)	CGC
JDH1601(*Psir5::gfp::let858.3-3'UTR*)	This article
JDH1602(*Psir5::inos-1::gfp::let858.3-3'UTR*)	This article
JDH1703(*gfp;daf-16(mgDf50)*)	This article
JDH1704(*inos-1::gfp;daf-16(mgDf50)*)	This article
JDH1705(*gfp;daf-18(ok480)*)	This article
JDH1706(*inos-1::gfp;daf-18(ok480)*)	This article
JDH1709(*gfp;akt-2(ok393)*)	This article
JDH1710(*inos-1::gfp;akt-2(ok393)*)	This article
JDH1711(*gfp;akt-1(ok525))*	This article
JDH1712(*inos-1::gfp;akt-1(ok525)*)	This article
KX15 (*ife-2(OK306)*)	CGC
MQ466(*pigv-1(qm34)*)	CGC
MQ887(*isp-1(qm150)*)	CGC
MT1083(*egl-8(n488)*)	CGC
N2 (Bristol)	CGC
NL5901(*pkIs2386 [unc-54p::alphasynuclein::yfp+ unc-119(+)]*)	Prof. Martin S. Denzel
PS2286(*lfe-2(sy326))*	CGC
PS3653(*ipp-5(sy605))*	CGC
RB1373(*gpdh-1(ok1558)*)	CGC
RB1813(*piki-1(ok2346))*	CGC
RB2547(*pink-1(ok3538))*	CGC
RB712(*daf-18(ok480))*	CGC
RM2221(*egl-8(md1971))*	CGC
TJ1052(*age-1(hx546))*	CGC
VC1024(*pdr-1(gk448))*	CGC
VC161(*mtm-6(ok330))*	CGC
VC199 (*sir-2.1(ok434)*)	CGC
VC204(*akt-2(ok393))*	CGC
VC870(*nhr-49(gk405)*)	CGC
VC963(*ppk-1(ok1411)/szT1 [lon-2(e678)] I; +/szT1 X*)	CGC

Supplementary Table 2

qPCR primers		
gpdh-1	Forward	TCAGAATTAGGGTGACAACGG
	Reverse	AGCGGATTTCACGTTGTAGG
Clone primers		
inos-1	Forward	CAGTGCGGCCGCATGAGCTCGGCCCAAGTTAATGGAAT
	Reverse	TCGAGGTACCGACTGAGATTTCG
RNAi primers		
C54E4.5	Forward	ATCGACTAGTGCTTATGAAGCCGACGGTAA
	Reverse	ATCGCTCGAGACCTGCCAAATGCATCCAC
pisy-1	Forward	ATCGACTAGTCGCCGAGAACAACAACAAT
	Reverse	ATCGCTCGAGGATGTCGATTACGGCCGAC
ppk-2	Forward	ATCGACTAGTAAAAAGGCAAGATTTTGGTGC
	Reverse	ATCGCTCGAGTCGTATGACGTCAGCGAGC
ZK795.1	Forward	ATCGACTAGTGCTATCAGAGACCGGCGATA
	Reverse	ATCGCTCGAGTGATAATTTTCAGCTTGTCCAGG

Supplementary Table 3

RC, indicates the rate-change to control. The initial animal number of each treatment is 35 animals per plate * 3 plate (FUdR+) or 20*5 (FUdR-).

P_T value is the P-value between CTL and Treatment (compound);

P_C value, two compounds of the same concentration;

P_M value, N2 and mutant or RNAi of the same Treatment.

BL is live bacteria; **BD** is killed bacteria.

Animals All indicates scored worm number in all biological replicates.

log rank test was used to calculate P value.

	Strain	Treat	Animals	P_T value	Mean ± SD (Day)	Mean RC (%)	Max ± SD (Day)	Max RC (%)	P_C value	Mean RC (%)	Max RC (%)	P_M value	Mean RC (%)	Max RC (%)	Animals All
1	N2_ev		43		17 ± 3		22 ± 0					CTL			81
	N2_*pisy-1* RNAi		43		14 ± 2		18 ± 1					4.30E-05	-14	-1	81

	Strain	Treat	Animals	P_T value	Mean ± SD (Day)	Mean RC (%)	Max ± SD (Day)	Max RC (%)	P_C value	Mean RC (%)	Max RC (%)	P_M value	Mean RC (%)	Max RC (%)	Animals All
2	N2_ev		38		15 ± 3		22 ± 1					CTL			38
	N2_C54E4.5 RNAi		51		16 ± 3		22 ± 1					7.27E-01	3	1	51

	Strain	Treat	Animals	P_T value	Mean ± SD (Day)	Mean RC (%)	Max ± SD (Day)	Max RC (%)	P_C value	Mean RC (%)	Max RC (%)	P_M value	Mean RC (%)	Max RC (%)	Animals All
4	N2		38		19 ± 2		24 ± 1					CTL			38
	mtm-6(ok330)		64		18 ± 2		22 ± 0					9.38E-04	-8	-7	64

	Strain	Treat	Animals	P_T value	Mean ± SD (Day)	Mean RC (%)	Max ± SD (Day)	Max RC (%)	P_C value	Mean RC (%)	Max RC (%)	P_M value	Mean RC (%)	Max RC (%)	Animals All
5	N2		50		20 ± 4		28 ± 0					CTL			121
	ppk-3(n2668)		35		16 ± 4		25 ± 0					1.04E-04	-23	-10	111

	Strain	Treat	Animals	P_T value	Mean ± SD (Day)	Mean RC (%)	Max ± SD (Day)	Max RC (%)	P_C value	Mean RC (%)	Max RC (%)	P_M value	Mean RC (%)	Max RC (%)	Animals All
7	N2_ev		43		17 ± 3		22 ± 0					CTL			81
	N2_*ppk-2* RNAi		30		17 ± 3		22 ± 0					6.05E-01	3	4	74

	Strain	Treat	Animals	P_T value	Mean ± SD (Day)	Mean RC (%)	Max ± SD (Day)	Max RC (%)	P_C value	Mean RC (%)	Max RC (%)	P_M value	Mean RC (%)	Max RC (%)	Animals All
8	N2		49		20 ± 2		25 ± 0					CTL			120
	pigv-1(qm34)		41		22 ± 3		31 ± 3					7.50E-02	6	23	128

	Strain	Treat	Animals	P_T value	Mean ± SD (Day)	Mean RC (%)	Max ± SD (Day)	Max RC (%)	P_C value	Mean RC (%)	Max RC (%)	P_M value	Mean RC (%)	Max RC (%)	Animals All
9	N2_ev		35		16 ± 3		22 ± 2					CTL			130
	N2_*pifk-1* RNAi		42		14 ± 3		22 ± 2					1.21E-01	-8	1	110

	Strain	Treat	Animals	P_T value	Mean ± SD (Day)	Mean RC (%)	Max ± SD (Day)	Max RC (%)	P_C value	Mean RC (%)	Max RC (%)	P_M value	Mean RC (%)	Max RC (%)	Animals All
11	N2		29		20 ± 4		27 ± 1								145
	piki-1(ok2346)		49		18 ± 2		23 ± 1					9.62E-04	-9	-14	142

	Strain	Treat	Animals	P_T value	Mean ± SD (Day)	Mean RC (%)	Max ± SD (Day)	Max RC (%)	P_C value	Mean RC (%)	Max RC (%)	P_M value	Mean RC (%)	Max RC (%)	Animals All
13	N2		49		20 ± 2		22 ± 2					CTL			255
	ppk-1(ok1411) $^{+/-}$		64		18 ± 2		22 ± 2					7.44E-05	-11	-11	362
	N2_ev		54		19 ± 2		23 ± 1					CTL			54
	N2_*ppk-1* RNAi		67		17 ± 3		23 ± 0					2.59E-01	-6	0	67

	Strain	Treat	Animals	P_T value	Mean ± SD (Day)	Mean RC (%)	Max ± SD (Day)	Max RC (%)	P_C value	Mean RC (%)	Max RC (%)	P_M value	Mean RC (%)	Max RC (%)	Animals All
14	N2		49		20 ± 2		25 ± 0					CTL			325
	unc-26(s1710)		67		24 ± 4		31 ± 1					7.24E-10	18	22	363

	Strain	Treat	Animals	P_T value	Mean ± SD (Day)	Mean RC (%)	Max ± SD (Day)	Max RC (%)	P_C value	Mean RC (%)	Max RC (%)	P_M value	Mean RC (%)	Max RC (%)	Animals All
15	N2		71		21 ± 2		26 ± 0					CTL			229
	age-1(hx546)		41		26 ± 8		39 ± 0					5.41E-08	25	50	120

	Strain	Treat	Animals	P_T value	Mean ± SD (Day)	Mean RC (%)	Max ± SD (Day)	Max RC (%)	P_C value	Mean RC (%)	Max RC (%)	P_M value	Mean RC (%)	Max RC (%)	Animals All
17	N2		71		21 ± 2		26 ± 0					CTL			412
	daf-18(ok480)		74		13 ± 2		16 ± 0					1.54E-35	-39	-37	331

	Strain	Treat	Animals	P_T value	Mean ± SD (Day)	Mean RC (%)	Max ± SD (Day)	Max RC (%)	P_C value	Mean RC (%)	Max RC (%)	P_M value	Mean RC (%)	Max RC (%)	Animals All
18	N2		71		20 ± 3		27 ± 2					CTL			142
	egl-8(md1971)		34		24 ± 5		31 ± 0					3.81E-05	15	15	99
	egl-8(n488)		55		22 ± 5		31 ± 0					4.04E-04	10	14	82
	N2		45		22 ± 3		29 ± 2					CTL			325
	egl-8(nj77)		37		28 ± 5		35 ± 1					1.03E-08	23	20	295

	Strain	Treat	Animals	P_T value	Mean ± SD (Day)	Mean RC (%)	Max ± SD (Day)	Max RC (%)	P_C value	Mean RC (%)	Max RC (%)	P_M value	Mean RC (%)	Max RC (%)	Animals All
19	N2		69		21 ± 3		28 ± 1					CTL			134
	lfe-2(sy326)		87		20 ± 2		24 ± 2					1.91E-04	9	19	161

	Strain	Treat	Animals	P_T value	Mean ± SD (Day)	Mean RC (%)	Max ± SD (Day)	Max RC (%)	P_C value	Mean RC (%)	Max RC (%)	P_M value	Mean RC (%)	Max RC (%)	Animals All
21	N2_ev		43		17 ± 3		22 ± 0					CTL			81
	N2_ZK795.1 RNAi		39		17 ± 3		23 ± 0					7.19E-01	0	-16	83

	Strain	Treat	Animals	P_T value	Mean ± SD (Day)	Mean RC (%)	Max ± SD (Day)	Max RC (%)	P_C value	Mean RC (%)	Max RC (%)	P_M value	Mean RC (%)	Max RC (%)	Animals All
23	N2		49		20 ± 2		25 ± 0					CTL			254
	ipp-5(sy605)		72		22 ± 2		26 ± 1					1.73E-04	9	5	339

	Strain	Treat	Animals	P_T value	Mean ± SD (Day)	Mean RC (%)	Max ± SD (Day)	Max RC (%)	P_C value	Mean RC (%)	Max RC (%)	P_M value	Mean RC (%)	Max RC (%)	Animals All
27	N2_ev		29		16 ± 4		25 ± 1					CTL			68
	N2_F46F11.1 RNAi		53		15 ± 2		19 ± 0					2.08E-02	-8	-21	77

28

Strain	Treat	Animals	P_T value	Mean ± SD (Day)	Mean RC (%)	Max ± SD (Day)	Max RC (%)	P_C value	Mean RC (%)	Max RC (%)	P_M value	Mean RC (%)	Max RC (%)	Animals All
N2_ev		29		16 ± 4		25 ± 1					CTL			68
N2_F30A10.3 RNAi		42		15 ± 2		20 ± 1					7.97E-02	-7	-17	88

29

Strain	Treat	Animals	P_T value	Mean ± SD (Day)	Mean RC (%)	Max ± SD (Day)	Max RC (%)	P_C value	Mean RC (%)	Max RC (%)	P_M value	Mean RC (%)	Max RC (%)	Animals All
N2		63		22 ± 3		28 ± 2					CTL			299
ttx-7(nj50)		48		20 ± 2		24 ± 1					2.00E-04	-8	-15	232

Strain	Treat	Animals	P_T value	Mean ± SD (Day)	Mean RC (%)	Max ± SD (Day)	Max RC (%)	P_C value	Mean RC (%)	Max RC (%)	P_M value	Mean RC (%)	Max RC (%)	Animals All
N2	CTL	50	CTL	20 ± 4	CTL	28 ± 0	CTL							304
~	M 50mM	32	2.56E-01	19 ± 4	-6	29 ± 1	3							262
~	MI 50mM	42	5.46E-03	23 ± 4	14	31 ± 1	8							292

Strain	Treat	Animals	P_T value	Mean ± SD (Day)	Mean RC (%)	Max ± SD (Day)	Max RC (%)	P_C value	Mean RC (%)	Max RC (%)	P_M value	Mean RC (%)	Max RC (%)	Animals All
gfp		55		18 ± 5		29 ± 1					CTL			466
inos-1::gfp		55		22 ± 6		32 ± 1					1.61E-03	19	11	447

Strain	Treat	Animals	P_T value	Mean ± SD (Day)	Mean RC (%)	Max ± SD (Day)	Max RC (%)	P_C value	Mean RC (%)	Max RC (%)	P_M value	Mean RC (%)	Max RC (%)	Animals All
N2		71		21 ± 2		26 ± 0					CTL			120
ppk-1(ok1411)+/-		93		19 ± 2		23 ± 0					1.02E-04	-7	-10	157
unc-26(s1710)		77		24 ± 3		31 ± 1					2.23E-08	12	17	144
age-1(hx546)		41		26 ± 8		39 ± 0					5.41E-08	25	50	70
daf-18(ok480)		74		13 ± 2		16 ± 0					1.54E-35	-39	-37	87
egl-8(md1971)		65		24 ± 3		30 ± 1					3.33E-11	15	16	128

Strain	Treat	Animals	P_T value	Mean ± SD (Day)	Mean RC (%)	Max ± SD (Day)	Max RC (%)	P_C value	Mean RC (%)	Max RC (%)	P_M value	Mean RC (%)	Max RC (%)	Animals All
N2	CTL	39	CTL	16 ± 3	CTL	23 ± 0	CTL				CTL			
~	MI 50mM	46	2.40E-03	19 ± 3	15	24 ± 0	5							
ppk-1(ok1411)+/-	CTL	54	CTL	13 ± 2	CTL	18 ± 0	CTL				1.86E-07	-20	-22	
~	MI 50mM	56	2.17E-02	14 ± 2	8	19 ± 1	5				3.21E-13	-25	-22	

Strain	Treat	Animals	P_T value	Mean ± SD (Day)	Mean RC (%)	Max ± SD (Day)	Max RC (%)	P_C value	Mean RC (%)	Max RC (%)	P_M value	Mean RC (%)	Max RC (%)	Animals All
N2	CTL	53	CTL	14 ± 4	CTL	24 ± 0	CTL				CTL			218
~	MI 50mM	67	9.80E-04	17 ± 5	19	28 ± 1	19							222
daf-18(ok480)	CTL	50	CTL	12 ± 2	CTL	18 ± 1	CTL				1.40E-03	-15	-23	177
~	MI 50mM	55	2.30E-01	12 ± 2	-4	16 ± 2	-8				2.95E-14	-32	-41	168

Strain	Treat	Animals	P_T value	Mean ± SD (Day)	Mean RC (%)	Max ± SD (Day)	Max RC (%)	P_C value	Mean RC (%)	Max RC (%)	P_M value	Mean RC (%)	Max RC (%)	Animals All
gfp		67		20 ± 4		27 ± 1		CTL			CTL			97
inos-1::gfp		58		23 ± 4		33 ± 1		8.92E-04	14	20				78
gfp;daf-18(ok480)		45		14 ± 2		20 ± 0		CTL			1.21E-15	-30	-26	70
inos-1::gfp;daf-18(ok480))		53		13 ± 2		19 ± 1		2.06E-01	-7	-5	2.47E-25	-43	-42	84

Strain	Treat	Animals	P_T value	Mean ± SD (Day)	Mean RC (%)	Max ± SD (Day)	Max RC (%)	P_C value	Mean RC (%)	Max RC (%)	P_M value	Mean RC (%)	Max RC (%)	Animals All
N2	CTL	39	CTL	16 ± 3	CTL	23 ± 0	CTL				CTL			93
~	MI 50mM	46	2.40E-03	19 ± 3	15	24 ± 0	5							93
daf-16(mgDf50)	CTL	57	CTL	12 ± 2	CTL	17 ± 0	CTL				3.68E-09	-24	-24	115
~	MI 50mM	73	8.55E-07	15 ± 2	18	19 ± 0	7				2.43E-13	-22	-22	129

Strain	Treat	Animals	P_T value	Mean ± SD (Day)	Mean RC (%)	Max ± SD (Day)	Max RC (%)	P_C value	Mean RC (%)	Max RC (%)	P_M value	Mean RC (%)	Max RC (%)	Animals All
N2	CTL	43	CTL	18 ± 4	CTL	26 ± 3	CTL				CTL			241
~	MI 50mM	35	1.27E-03	22 ± 5	20	29 ± 1	12							213
pink-1(ok3538)	CTL	42	CTL	16 ± 4	CTL	25 ± 0	CTL				5.24E-02	-12	-4	205
~	MI 50mM	35	4.76E-01	16 ± 5	4	27 ± 3	7				4.77E-04	-23	-8	169

Strain	Treat	Animals	P_T value	Mean ± SD (Day)	Mean RC (%)	Max ± SD (Day)	Max RC (%)	P_C value	Mean RC (%)	Max RC (%)	P_M value	Mean RC (%)	Max RC (%)	Animals All
N2_BD_FUdR+	CTL	33	CTL	19 ± 2	0	24 ± 0	0							64
~	MI 1mM	29	5.45E-01	18 ± 2	-4	24 ± 1	-1							62
~	MI 3mM	35	6.76E-01	19 ± 2	0	24 ± 1	1							59
~	MI 10mM	26	5.16E-01	19 ± 2	1	24 ± 1	1							63
~	MI 30mM	35	2.68E-01	20 ± 3	4	26 ± 3	9							82
~	MI 100mM	32	1.45E-03	21 ± 3	11	26 ± 1	10							82
~	MI 200mM	44	1.20E-05	22 ± 3	15	28 ± 0	15							98
~	MI 300mM	55	1.41E-07	23 ± 3	18	30 ± 4	23							110
~	MI 400mM	66	4.46E-13	24 ± 3	25	31 ± 4	31							145
~	MI 500mM	81	1.68E-19	25 ± 4	31	33 ± 2	36							147
~	MI 600mM	65	8.63E-14	24 ± 3	26	29 ± 1	20							125
~	MI 700mM	38	3.84E-09	24 ± 4	23	30 ± 1	24							77

Strain	Treat	Animals	P_T value	Mean ± SD (Day)	Mean RC (%)	Max ± SD (Day)	Max RC (%)	P_C value	Mean RC (%)	Max RC (%)	P_M value	Mean RC (%)	Max RC (%)	Animals All
N2_BD_FUdR+	CTL	86	CTL	20 ± 2	CTL	24 ± 1	CTL							86
~	IP6 1mM	100	3.00E-01	21 ± 2	2	24 ± 1	1							100
~	IP6 3mM	96	3.20E-02	21 ± 2	3	25 ± 0	2							96
~	IP6 10mM	111	3.23E-10	22 ± 3	10	27 ± 1	12							111
~	IP6 30mM	102	1.10E-14	23 ± 3	14	29 ± 1	19							102
~	IP6 50mM	97	8.62E-12	23 ± 3	11	27 ± 1	13							97

Strain	Treat	Animals	P_T value	Mean ± SD (Day)	Mean RC (%)	Max ± SD (Day)	Max RC (%)	P_C value	Mean RC (%)	Max RC (%)	P_M value	Mean RC (%)	Max RC (%)	Animals All
N2	CTL	39	CTL	16 ± 3	CTL	23 ± 0	CTL				CTL			93
~	MI 50mM	46	2.40E-03	19 ± 3	15	24 ± 0	5							93
akt-1(ok525)	CTL	46	CTL	22 ± 3	CTL	31 ± 3	CTL				2.83E-10	35	33	97

~	MI 50mM	52	4.12E-02	24 ± 4	7	33 ± 2	9				6.93E-10	26	37	111
akt-2(ok393)	CTL	54	CTL	20 ± 4	CTL	30 ± 4	CTL				3.96E-04	19	29	96
~	MI 50mM	50	3.88E-03	22 ± 5	11	31 ± 3	3				1.93E-05	15	26	74

Strain	Treat	Animals	P_T value	Mean ± SD (Day)	Mean RC (%)	Max ± SD (Day)	Max RC (%)	P_C value	Mean RC (%)	Max RC (%)	P_M value	Mean RC (%)	Max RC (%)	Animals All
N2	CTL	43	CTL	18 ± 4	CTL	26 ± 3	CTL				CTL			83
~	MI 50mM	35	1.39E-06	23 ± 3	29	30 ± 1	13							77
pdr-1(gk448)	CTL	42	CTL	19 ± 2	CTL	25 ± 1	CTL				4.74E-01	5	-4	74
~	MI 50mM	35	3.58E-02	21 ± 3	8	26 ± 1	5				1.24E-03	-11	-10	70

Strain	Treat	Animals	P_T value	Mean ± SD (Day)	Mean RC (%)	Max ± SD (Day)	Max RC (%)	P_C value	Mean RC (%)	Max RC (%)	P_M value	Mean RC (%)	Max RC (%)	Animals All
N2	CTL	47	CTL	19 ± 3	CTL	25 ± 2	CTL				CTL			109
~	MI 50mM	50	1.24E-03	22 ± 3	11	29 ± 1	17							115
gpdh-1(ok1558)	CTL	59	CTL	19 ± 4	CTL	25 ± 1	CTL				8.95E-01	-2	1	123
~	MI 50mM	40	3.86E-03	22 ± 3	15	29 ± 3	13				8.42E-01	0	-1	100
