# Supplementary material for: The precursor of PI(3,4,5)P3 alleviates aging by activating daf-18(Pten) and independent of daf-16
Source: Nat Commun. 2020 Sep 8;11:4496. doi: 10.1038/s41467-020-18280-4 (PMC7479145; doi:10.1038/s41467-020-18280-4)
Supplement: Supplementary file 3 — Description of Additional Supplementary Information [file 41467_2020_18280_MOESM3_ESM.pdf]

Description of Additional Supplementary Files

File Name: Supplementary Data 1

Description: Aging up and down-regulated genes in worm and mouse, respectively. ENSEMBL Gene ID (WORM BASE GENE ID for worm), Gene Symbol, ENTREZID (Sequence name for worm). The worm dataset was determined by $|log_2$-fold change$|>1$ and the mouse dataset was determined by $|log_2$-fold change$|>0.5$ and $p<0.05$ given by DEseq2.

File Name: Supplementary Data 2

Description: The expression changes of all ENSEMBL genes upon MI treatment of 12 month-old mice. ENSEMBL Gene ID, Gene Symbol, mean expression of all samples (baseMean), log_2-fold change, standard error of log_2-fold change (lfcSE), Wald statistic (stat), *p* value and adjusted *p* value as determined by DEseq2 are shown from left to right.
